# The DNA2 nuclease/helicase is an estrogen-dependent gene mutated in breast and ovarian cancers

**DOI:** 10.18632/oncotarget.2414

**Published:** 2014-11-01

**Authors:** Carmit Strauss, Maya Kornowski, Avraham Benvenisty, Amit Shahar, Hadas Masury, Ittai Ben-Porath, Tommer Ravid, Ayelet Arbel-Eden, Michal Goldberg

**Affiliations:** ^1^ Department of Genetics, Alexander Silberman Institute of Life Sciences, Hebrew University of Jerusalem, Jerusalem, 91904, Israel; ^2^ Department of Developmental Biology and Cancer Research, IMRIC, Hebrew University-Hadassah Medical School, Jerusalem, 91120, Israel; ^3^ Department of Biochemistry, Alexander Silberman Institute of Life Sciences, Hebrew University of Jerusalem, Jerusalem, 91904, Israel; ^4^ Department of Medical Laboratory Sciences, Hadassah Academic College, Jerusalem, 91010, Israel

**Keywords:** Estrogen-dependent cancers, DNA helicases, DNA nucleases, DNA2, estrogen, DNA damage response

## Abstract

Genomic instability, a hallmark of cancer, is commonly caused by failures in the DNA damage response. Here we conducted a bioinformatical screen to reveal DNA damage response genes that are upregulated by estrogen and highly mutated in breast and ovarian cancers. This screen identified 53 estrogen-dependent cancer genes, some of which are novel. Notably, the screen retrieved 9 DNA helicases as well as 5 nucleases. DNA2, which functions as both a helicase and a nuclease and plays a role in DNA repair and replication, was retrieved in the screen. Mutations in DNA2, found in estrogen-dependent cancers, are clustered in the helicase and nuclease domains, suggesting activity impairment. Indeed, we show that mutations found in ovarian cancers impair DNA2 activity. Depletion of DNA2 in cells reduces their tumorogenicity in mice. In human, high expression of DNA2 correlates with poor survival of estrogen receptor-positive patients but not of estrogen receptor-negative patients. We also demonstrate that depletion of DNA2 in cells reduces proliferation, while addition of estrogen restores proliferation. These findings suggest that cells responding to estrogen will proliferate despite being impaired in DNA2 activity, potentially promoting genomic instability and triggering cancer development.

## INTRODUCTION

A prominent factor in tumorigenesis is genomic instability, which involves changes in nucleic acid sequences, structural chromosomal rearrangements, and whole chromosome copy-number alterations. Failures in DNA replication and in DNA damage response (DDR) are common causes of genomic instability [1,2].

Helicases are motor proteins that unwind structured nucleic acid. They are important for maintenance of genomic stability and implicated in DNA repair, replication, recombination transcription and chromosome segregation [3,4]. Many DNA helicases are overexpressed in cancer cells, enabling these cells to deal with replicative lesions that arise in highly proliferative states and with DNA damage caused by radiation or chemotherapy, which frequently relies on administration of DNA-damaging agents. On the other hand, loss of helicase functions promotes genomic instability and can result in carcinogenesis [3].

Numerous nucleases play a role in DNA double-strand break (DSB) repair as they are required for DNA end resection, which is an essential step in DSB repair by homologous recombination (HR). DSBs are considered the most hazardous type of DNA damage, since impairment in the response to these lesions may result in genomic instability which is a hallmark of cancer cells [5–8].

Estrogen-dependent cancers (breast, uterine, and ovarian cancers) are leading causes of mortality in women [9]. Estrogens, which are the primary female sex hormones, are tumorigenic since they stimulate cell proliferation and induce DNA damage. They stimulate proliferation by binding to the estrogen receptors (ERs), which are transcription factors that modulate the expression of various genes involved in different processes, including cell proliferation [10]. In addition, estrogens are genotoxic agents since they are converted to metabolites that can react with DNA to form depurinating adducts, which are released from DNA to generate apurinic sites [11]. Also, transcription regulation by estrogens, which occurs via binding to the ERs, has been suggested to induce DSB formation at the sites of estrogen inducible genes [12].

Here we conducted a computational screen to reveal novel genes involved in estrogen-dependent cancers. We aimed for genes that are upregulated by estrogen, play a role in the DDR and are highly mutated in breast and ovarian cancers. This screen identified 53 estrogen-dependent cancer genes, some of which are novel. Notably, the screen retrieved 9 DNA helicases as well as 5 nucleases. We focused on the involvement of the helicase and nuclease DNA2 in estrogen-dependent tumorigenesis. We discovered that mutations in DNA2, found in estrogen-dependent cancers, are clustered in its helicase and nuclease domains and impair DNA2 activity. Depletion of DNA2 in cells reduces the tumorogenity of these cells in mice. In human, high expression of DNA2 correlates with poor prognosis of ER-positive breast cancer patients. Moreover, cells depleted of DNA2 cease to proliferate, while addition of estrogen restores proliferation.

## RESULTS

### A bioinformatic screen for novel estrogen-dependent cancer genes

Estrogen-regulated genes are often central in the proliferation and function of estrogen-dependent cancer cells [13]. Additionally, impairment in the DDR may result in genomic instability [14]. In light of this, we screened for DDR genes that are regulated by estrogen and highly mutated in estrogen-dependent cancers. The initial step in the bioinformatic screen was to analyze for changes in gene expression profiles due to treatment with estrogen. The data was obtained from Gene Expression Omnibus (GEO; http://www.ncbi.nlm.nih.gov/geo) and included studies that determined the expression profiles of MCF7 cells, which are ER-positive breast cancer cells, that were mock-treated or treated with estradiol (E2; the primary type of estrogen). Studies in which estrogen addition resulted in upregulation of at least 5 out of 6 genes from a well characterized signature for E2-upregulated genes (GREB1, MYB, MYBL1, MYBL2, MYC and PGR [15]) were considered valid. Two studies, which analyzed treatments with E2 for 3, 6, or 24h, had these criteria [16,17]. We calculated, for each gene present in these studies, the ratio of expression between cells treated with E2 and mock-treated control cells. We determined that a gene is upregulated by estrogen if this ratio of expression was above 2 in at least one experiment. This step retrieved 1280 genes (Supplementary Table S1).

**Figure 1:**
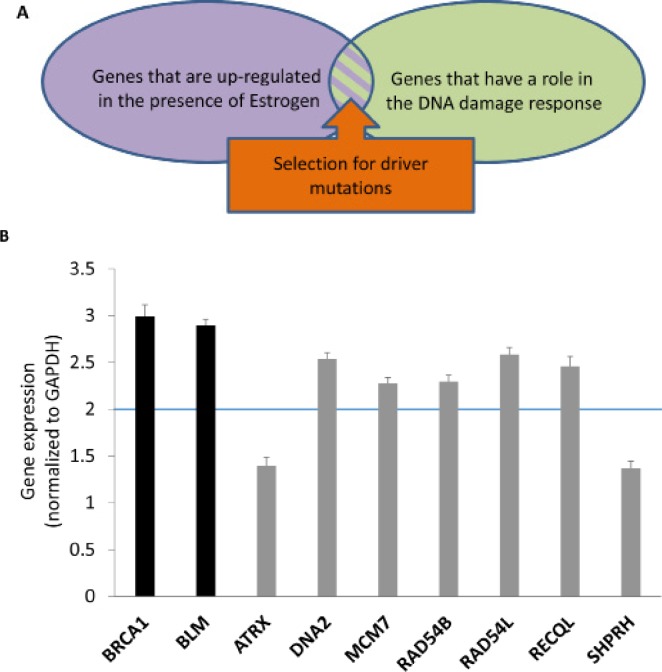
Upregulation of helicases and nucelases that are highly mutated in breast or ovarian cancers by estrogen **(A)** screen architecture. The screen scored for genes that are upregulated by estrogen, part of the DNA damage response and highly mutated in estrogen-dependent cancers. **(B)** MCF7 cells were treated with E2 (25nM) or mock treated for 24h. Expression levels of indicated genes were detected by real-time PCR. Gene expression levels were normalized to GAPDH. Black bars represent control genes. Gray bars represent genes of interest.

DDR genes responding to estrogen are good candidates for involvement in estrogen-dependent cancers since the genes engaged in familial breast and ovarian cancers participate in different aspects of the DDR [18,19]. Hence, we filtered the genes upregulated by estrogen to genes that cluster as DNA repair using the DAVID functional annotation clustering tool (http://david.abcc.ncifcrf.gov; GO_TERM_BP_5; Figure 1A). This narrowed the number of genes of interest to 69 (Supplementary Tables S2 and S3).

Somatic mutations found in cancer can be “driver” mutations that trigger tumorigenesis, or “passenger” mutations that are retained by chance during repeated rounds of cell division and clonal expansion and do not provide an advantage to the tumor [20,21]. The probability for receiving the number of mutations found in tumors in a particular candidate gene was tested (Figure 1A), taken into account the size of the gene and the tissue specify. We analyzed the dbGaP breast and ovarian cancer databases (database of Genotypes and Phenotypes; http://www.ncbi.nlm.nih.gov/gap), based on the estimation that in a tumor the rate of non-silent spontaneous mutations in coding regions is 0.33 per Mb in breast cancers and 0.44 per Mb in ovarian cancers (average of 0.33 and 0.55 per Mb in breast and colorectal cancers, respectively; [20,22]). 53 E2-upregulated DDR genes were significantly mutated in either breast or ovarian cancers (p-value<0.05; Supplementary Tables S2 and S3). The mutation enrichment was observed in 45 and 26 E2-upregulated DDR genes in breast and ovarian cancers, respectively (Supplementary Tables S2 and S3). Previously reported estrogen-dependent cancer genes including classical breast and ovarian cancer genes (e.g. BRCA1, BRCA2 and CHK2) as well as novel genes were scored in the screen (Table 1 and Supplementary Tables S2 and S3). Remarkably, the screen scored for a high proportion of genes encoding helicases and nucleases (helicases- 17%: ATRX, BLM, BRIP1, DNA2, MCM7, RAD54B, RAD54L, RECQL1, and SHPRH; nucleases- 7.55%: DNA2, EXO1, FEN1, GEN1, and MRE11A; Table 1 and Supplementary Tables S2 and S3). 7 of the retrieved helicases and nucleases were not previously reported to contain mutations or polymorphisms in association with breast or ovarian cancers (Table 1, genes marked with an asterisk). Next we tested if these 7 helicases and nucleases are upregulated by estrogen, as implied from the gene expression profiles from the bioinformatic screen. MCF7 cells were incubated with E2 for 24h and changes in mRNA levels compared to control were analyzed by real-time PCR. As a control for induction of E2, expression of BLM and BRCA1, which are known to be upregulated by estrogen [23,24], was tested. We validated that 5 out of the 7 novel estrogen-dependent cancer genes are upregulated by estrogen in MCF7 cells (Figure 1B).

DNA2 was retrieved in the screen (Table 1). Since DNA2 functions as both a helicase and a nuclease we continued studying its involvement in estrogen-dependent cancers. DNA2 is involved in maturation of Okazaki fragments during DNA replication, in preventing stalled replication forks from reversing, and in DNA repair [25–29]. DNA2 may therefore be implicated in cancer development in a multifaceted manner. Mutations in DNA2 can act as driver mutations that promote tumorigenesis through induction of DNA damage. On the other hand, high expression of DNA2 may increase its helicase activity and thus facilitate coping with replicative lesions arising in highly proliferative states and with DNA damage caused by radiation or chemotherapy [3].

### Mutations in the DNA2 gene, found in estrogen-dependent cancer cases, are clustered in the helicase and nuclease domains

**Figure 2:**
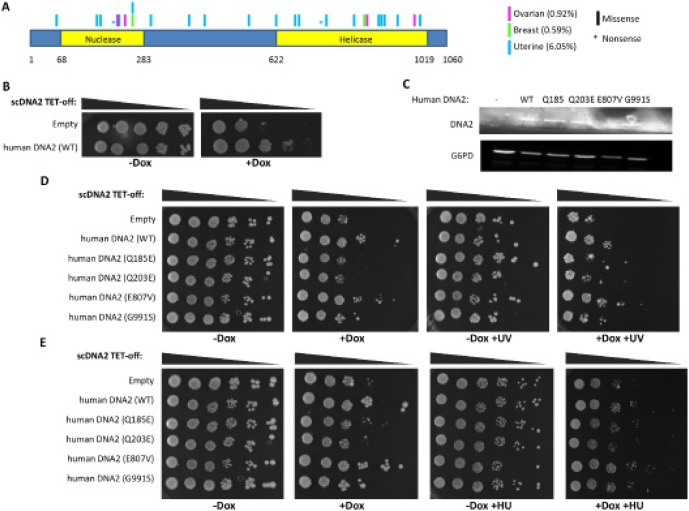
Somatic mutations found in cancer cases impair the activity of DNA2 **(A)** schematic representation of the position of non-silent somatic mutations in DNA2 associated with estrogen dependent cancers. The data was obtained from the COSMIC database. The cartoon represents mutation type (shape), tissue (color) and position in the encoded protein. Percentage in brackets represents mutation frequency of DNA2 in the relevant cancer. **(B)** TET-off scDNA2 yeast cells, expressing human DNA2 or a control vector, were seeded in serial dilutions and grown at 30° for 72 hours on a control media (-Dox) or with Dox (+Dox). **(C)** Western blot analysis for DNA2 protein expression of the different human DNA2 constructs in the TET-off scDNA2 strain was tested. Control for protein loading is endogenous G6PD. **(D,E)** TET-off scDNA2 yeast cells, expressing human DNA2, WT or mutants, or a control vector, were seeded in serial dilutions and grown at 30° for 96 (D) or 144 (E) hours on a control media (-Dox) or with Dox (+Dox). The plates were either treated or mock-treated with UV (D) or supplemented with HU (E).

The distribution of mutations in a particular gene in cancer cases may imply if these mutations affect gene function and by that trigger cancer formation. We analyzed the cosmic database (http://cancer.sanger.ac.uk/cancergenome/projects/cosmic/) for mutations in the DNA2 gene in estrogen-dependent cancer cases. Remarkably, most of the somatic mutations were found to be clustered in the helicase or nuclease domains of DNA2 (Figure 2A). This suggests that the cancer-related mutations in DNA2 lead to inactivation or impairment of DNA2 activity, which is known to be essential for the maintenance of genomic stability [30,31].

**Table 1:** List of selected genes obtained from the screen for DDR genes that are upregulated by estrogen and are highly mutated in breast and/or ovarian cancers Gene column- genes retrieved in the screen. In each sub-group (classical, helicases and nucleases) the genes are organized in alphabetical order. Ratio column- gene expression levels in E2-treated MCF7 cells divided by mock treatment. dbGaPOvarian and dbGaP-Breast columns- P-values of the probability that the mutations found in a candidate gene in the dbGaP database are random. Asterisks represent genes that were not reported previously to contain mutations or polymorphism in association to breast or ovarian cancers.

		Gene	Ratio (+E2/−E2)	dbGAP-Ovarian	dbGAP-Breast
Classic breast/ovarian cancer genes		BRCA1	2.80823	<10E-08	<10E-08
		BRCA2	3.06944	<10E-08	<10E-08
		CASC5	2.84378	0.84753	1.00E-08
		CHEK2	2.00318	<10E-08	7.01E-05
		RAD51	3.12499	0.03371	0.00032
		TOP2A	3.10101	<10E-08	0.22244
	Helicases	ATRX*	2.44879	0.31567	1.40E-07
		BLM	2.30394	0.0002	<10E-08
		BRIP1	3.01241	0.36822	1.20E-07
		MCM7*	2.72645	0.73956	<10E-08
		RAD54B*	2.56894	0.76498	<10E-08
		RAD54L*	2.93684	0.0081	0.02954
		RECQL*	2.39143	0.15134	0.01652
		SHPRH*	2.23607	0.01896	0.00054
		DNA2*	2.15806	6.60E-06	0.09365
Nucleases		EXO1	4.93706	0.01471	0.04652
		FEN1	2.46895	0.04676	0.09445
		GEN1	2.10712	0.02004	0.79797
		MRE11A	2.12168	0.73797	0.00042

### Somatic mutations in DNA2 occurring in ovarian cancers impair the activity of DNA2

Searching the dbGaP database we found four missense mutations in the DNA2 gene in ovarian cancer cases. Two mutations are located at the nuclease domain and two at the helicase domain (Figure 2A, magenta and Supplementary Table S4). The mutations in the nuclease domain occurred at less conserved residues compared to the mutations in the helicase domain, which occurred at residues conserved even in the yeast *Saccharomyces cerevisiae* (Supplementary Table S4). In order to gain insight into how these mutations contribute to cancer development, we developed a system in yeast to determine whether these mutations abrogate DNA2 activity. Yeast DNA2 (scDNA2) is well characterized and depletion of scDNA2 is lethal. It was demonstrated that the helicases human DNA2 and human BLM can suppress the growth defects of the temperature sensitive dna2-1 strain, which is mutated in scDNA2. Moreover, a mutated form of human BLM, which contains a mutation (K695T) that inactivates its helicase activity, failed to complement the growth defects of the dna2-1 strain [32]. This implies that an active helicase is required in order to overcome the growth defects resulting from the impairment of scDNA2. An addition benefit of the yeast system is that it enables to express different forms of human DNA2 (WT and mutants) at relatively equal levels. The TET-off system was used to deplete scDNA2 expression. We introduced human DNA2 to the TET-off scDNA2 strain (Hughes collection [33]) and incubated the yeast cells in the presence of Doxycycline (Dox) to inhibit scDNA2 synthesis. Human DNA2 partially complemented the lethal phenotype associated with the depletion of scDNA2 (Figure 2B). Next, we tested the ability of human DNA2, harboring the mutations found in ovarian cancer cases (Figure 2A, magenta and Supplementary Table S4), to complement the TET-off scDNA2 strain growth defect. Since changes in the expression levels of DNA2 may affect the yeast growth phenotype, we verified by Western-blot analysis that the expression levels of the different forms of human DNA2 (WT or mutated forms) are comparable (Figure 2C). The growth defect induced by scDNA2 depletion by DOX was not complemented by two mutated forms of human DNA2 (Q185E and Q203E; Figure 2D) and was partially complemented by the G991S mutant. The E807V mutant complemented the growth of the scDNA2 strain similarly to WT human DNA2 (Figure 2D).

DNA2 plays a role in DNA repair and replication [25–29], hence the effect of the mutated variants of human DNA2 was analyzed under DNA damage and replication stresses. The TET-off scDNA2 strains expressing WT or mutated forms of human DNA2 were subjected to UV radiation or incubated on plates with hydroxyurea (HU) or MMS. Replication stress, induced by HU, highly impaired cell growth even in the presence of scDNA2 (Figure 2E; compare -Dox and -Dox +HU plates). The expression of different forms of DNA2 between cells growing with and without HU had a similar effect on the growing of yeast depleted for scDNA2 (compare Figure 2E +Dox to +Dox+HU). Notably, testing the ability of the human DNA2 mutants to complement the scDNA2 phenotype upon DNA damage induction, due to UV radiation or MMS supplementation, demonstrated that the G991S mutant also impairs DNA2 activity, as was already indicated without DNA damage induction for the Q185E and Q203E mutants (Figure 2D and Supplementary Figure S1). The discovery that mutations, which occurred in cancer cases did not complement the yeast phenotype at all, or partially complemented the phenotype compared to the WT human DNA2, demonstrates that these mutations impair DNA2 activity (either full or partial impairment) [32].

### Depletion of DNA2 in human MDA-MB-435/GFP cells inhibits xenograft growth in mice

**Figure 3:**
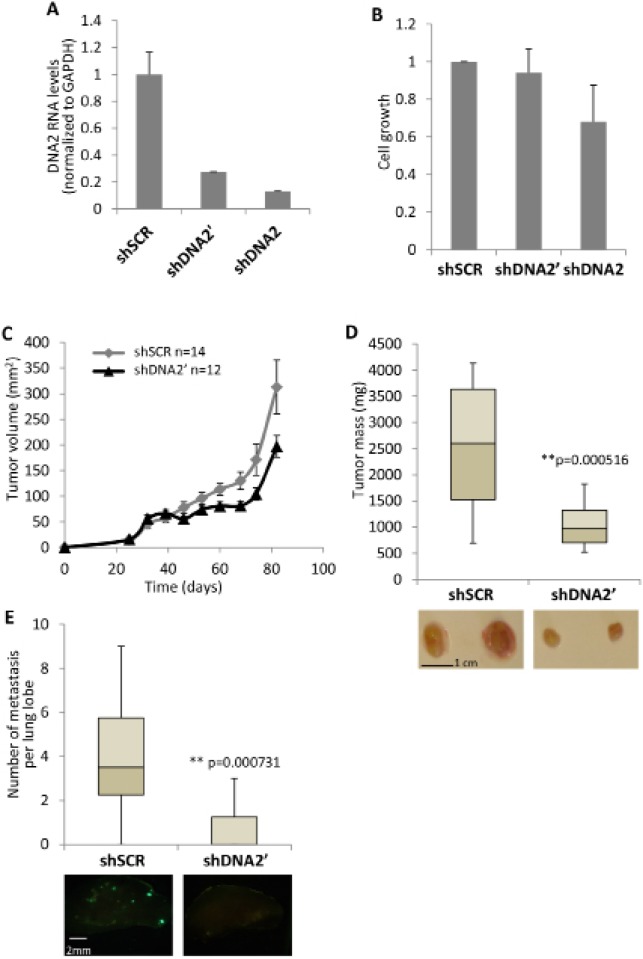
Downregulation of DNA2 results in slower cell and tumor growth **(A, B)** MDA-MB-435 cells were infected with two shRNAs directed against DNA2 (shDNA2 and shDNA2′) or with a control shRNA (shSCR). Downregulation of DNA2 expression was analyzed by real-time PCR (A). cells were plated at equal numbers in a 6-well plate. 5 days later, cells were fixed and cellular growth was tested (B). **(C)** MDA-MB-435/GFP cells, down-regulated for DNA2 or control, were injected to female mice mammary fat pad. 10^6^ cells were used for each injection (day 0). From day 25 and forward, tumors were measured and tumor volume was calculated approximately every 7 days. **(D)** 82 days after the injection mice were sacrificed and the tumors were taken out and weighed. Below, pictures of representative tumors from the different treatments. **(E)** lungs were harvested from the mice described in D. The large lobe from every lung was analysed from both sides for metastases by florescent microscopy. Lumps above the size 100 μm^2^ were counted. Below, pictures of representative lobes from the different treatments. The whiskers in the box plots represent the minimum and maximum values of all data (D and E).

Downregulation of DNA2 in MCF7 and U20S cell lines results in reduced cell proliferation ([31,34] and Supplementary Figure S2) and in U2OS cells it also induces cellular senescence [34]. The effect of DNA2 downregulation on the aggressive MDA-MB-435 cell line was tested. We infected MDA-MB-435 cells that stably express GFP (MDA-MB-435/GFP) with two shRNAs directed against DNA2 (shDNA2 and shDNA2′) and a control shRNA (shSCR), and verified the depletion of DNA2 by real-time PCR (Figure 3A). Next, we analyzed the cell growth of these 3 cell populations. While MDA-MB-435/GFP infected with shDNA2 grow slower than the control cells, the growth of cells infected with shDNA2′ was relatively similar to the control (Figure 3B). Since the growing rate of MDA-MB-435/GFP cells infected with shDNA2′ was comparable to that of the control, this cell line was used to analyze whether DNA2 supports tumor growth. MDA-MB-435/GFP cells infected with shDNA2′, or with the control shSCR, were injected bilaterally into the #4 mammary glands of NOD/SCID female mice. Tumor formation in mice was monitored for 12 weeks and tumor volume was measured (Figure 3C). Reduced tumor weight was obtained in tumors derived from cells depleted of DNA2 as compared to the control (Figure 3D). Next, the lungs from the sacrificed mice were dissected and metastasis formation was evaluated by analyzing for cells expressing GFP, which originated from the MDA-MB-435/GFP cells. Lung metastasis formation was almost prevented when DNA2-depleted cells were injected (Figure 3E). These results indicate that DNA2 is required for xenograft growth and suggest that its expression in breast cancer can be tumor promoting. Inhibition of metastasis formation may indicate impairment in metastasis formation (e.g. migration, local invasion or survival in the circulation) but may also be a result of slower growth rate, resulting in lower detection capacity of the metastasis.

### DNA2 expression levels are altered in cancer cells

**Figure 4:**
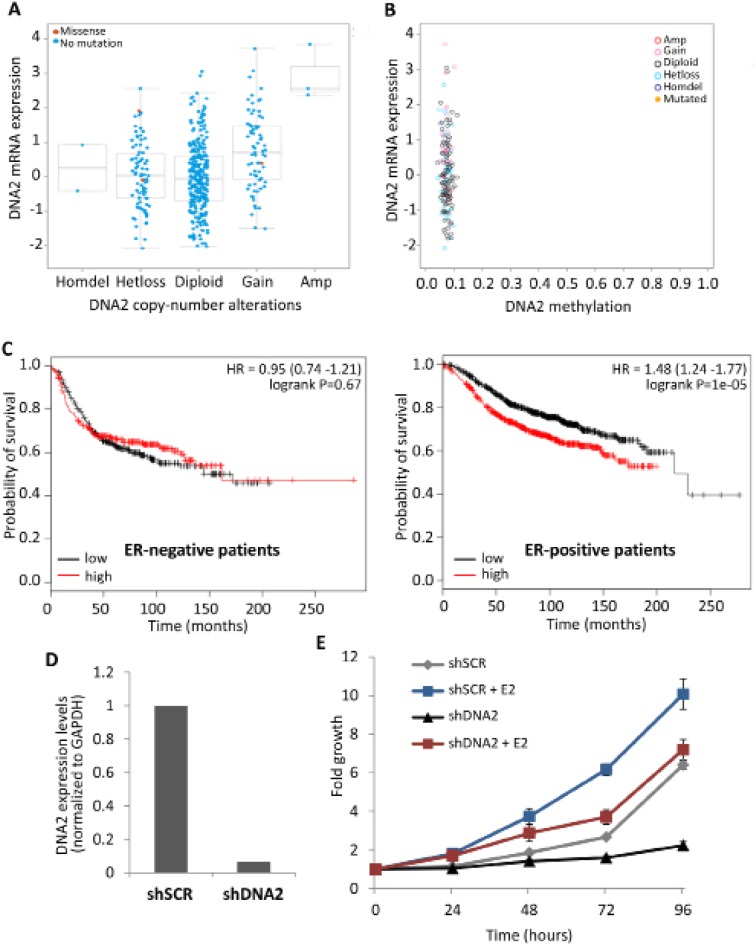
Higher DNA2 expression levels correlates with poor prognosis in ER-positive tumors **(A, B)** DNA2 expression levels in 852 breast carcinomas (TCGA database) were analyzed using cBioPortal, and compared to CNA of DNA2 (A) or to the methylation levels of DNA2 in the same tumors (B). The whiskers in the box plot represent the 10^th^ and the 90^th^ percentile. **(C)** Kaplan-Meier (KM) overall survival curves according to the levels of DNA2 expression (black curve = 50% low expression; red curve = 50% high expression) were analyzed in ER-positive and ER-negative breast cancer patients. Left, ER-positive tumors (1,767 patients). Right- ER-negative tumors (668 patients). **(D, E)** MCF7 cells were infected with shDNA2 or with a control (shSCR), and grown in phenol red-free media with charcoal cleaned serum. Cells were plated in a 96-well plate, 24 h prior to the experiment. E2 (final concentration 10 μM) or ethanol were supplemented at time 0h. Expression levels of DNA2 were tested at time 0 by real-time PCR normalized by the expression of GAPDH (E). Cells were fixed at indicated time points and cellular growth was measured and compared to day 0 (F).

Since misregulation of helicase expression is a common feature of carcinogenesis [3] we examined, *in silico*, the expression levels of DNA2 in different types of estrogen-dependent cancers, using the cBioPortal for Cancer Genomics [35]. We matched DNA2 expression levels to copy-number alterations (CNA) in the DNA2 locus and found variation in the expression levels of different tumors, including those having a similar CNA pattern (Figure 4A and Supplementary Figure S3A). Higher expression of DNA2 is correlated with gain or amplification of the DNA2 locus (Figure 4A and Supplementary Figure S3A). The variation in the expression levels of DNA2 between cancer cells that have a similar DNA2 CNA pattern could be due to differences in the methylation levels of the DNA2 locus. We did not find a correlation between the expression levels and methylation levels of DNA2 (Figures 4B and Supplementary Figure S3B). These results highlight that changes in DNA2 expression are a common feature of tumors and that CNA can partially explain this variation.

### High expression level of DNA2 correlates with poor survival of ER-positive breast cancer patients

Kaplan-Meier (KM) curves, which are defined as the probability of surviving for a given length of time, show that in patients with breast cancer, DNA2 expression is inversely correlated with the duration of overall patient survival ([34] and Supplementary Figure S3C). We divided the breast cancer patients according to the ER expression status of their tumors and examined the KM curves obtained for ER-positive and ER-negative subgroups. Notably, the KM curves indicate that high DNA2 expression is significantly correlated with poor survival of the ER-positive patients (Figure 4C). Notably, such correlation was not detected for the ER-negative subgroup (Figure 4C). This implies that DNA2 overexpression significantly provides inferior survival only in cells responding to estrogen and that there is interplay between DNA2 and estrogen. Higher expression levels of DNA2 in tumor cells may attenuate the replication stress and DNA damage accumulation associated with cancer.

### Attenuation of cell growth due to depletion of DNA2 is reduced by estrogen

The interplay between DNA2 and estrogen was further studied by analyzing the effect of estrogen on the proliferation of MCF7 cells downregulated for DNA2. We infected MCF7 cells with two shRNAs against DNA2 (shDNA2 and shDNA2′) or with a control (shSCR) and confirmed downregulation of DNA2 using real-time PCR (Figure 4D and Supplementary Figure S3D). We grew the cells in media containing charcoal-stripped serum (no estrogen) and supplemented with E2 (estrogen) or ethanol (control). Cells downregulated for DNA2 that were grown in the absence of estrogen grew poorly (Figure 4E and Supplementary Figure S3E). Remarkably, addition of E2 to the media resulted in accelerated cell growth (Figure 4E and Supplementary Figure S3E). These results provide an explanation for the survival of cells that contain mutations in DNA2 in tissues that are located in the estrogen niche.

## DISCUSSION

Here we conducted a computational screen to reveal novel genes involved in estrogen-related cancers. First, we searched for genes that are upregulated by estrogen and play a role in the DDR. Involvement in the DDR was an important parameter in our screen since the genes found to be involved in familial breast and ovarian cancers participate in different aspects of the DDR [18,19]. Moreover, components of the DDR are potential drug targets for reduction of cancer cell viability and small molecule inhibitors that target the activity of different DDR members, such as PARP, DNA-PK, and ATM, are currently under development [2]. The next step in the screen was to score for genes that are highly mutated in estrogen-related cancers out of the DDR genes that are upregulated by estrogen. These criteria retrieved 53 DDR genes that are upregulated by estrogen and highly mutated in breast and ovarian cancers. These genes included established breast and ovarian cancer genes as well as genes that are potential estrogen-dependent cancer genes. Notably, there was an enrichment for helicases (17%) and nucleases (7.5%) among the retrieved genes. We focused on DNA2 that encodes a protein with both helicase and nuclease activities. Recently, Peng et al demonstrated that DNA2 expression is significantly increased in cancers and its expression correlates with patient outcome [34]. In our study we elaborated on the role of DNA2 in estrogen-dependent cancers and showed that DNA2 expression is not only increased but also reduced in cancer cells. Moreover, we showed that mutations in DNA2 frequently appear in tumors and that they are clustered in its helicase and nuclease domains, suggesting that these mutations impair the activity of DNA2. Indeed, we demonstrated that mutations from ovarian cancer cases impair DNA2 activity. We also found that depletion of DNA2 in cells reduces tumorigenicity of these cells in mice and that in human, high expression of DNA2 correlates with poorer survival of ER-positive patients. Moreover, our data reveals that while cells depleted of DNA2 cease proliferation, addition of estrogen restores proliferation.

### Helicases and cancer

**Figure 5:**
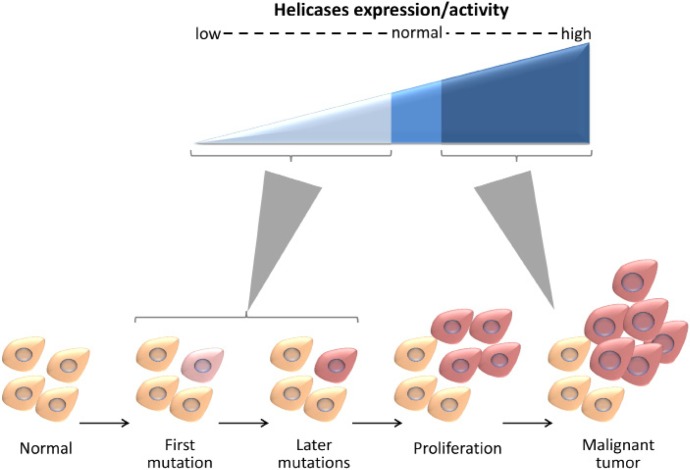
DNA helicases affect carcinogenesis in a multifaceted manner Low expression or impairment in the activity of helicases contribute to genomic instability and may trigger cancer development. High expression of helicases facilitates coping with replicative lesions that arise in highly proliferative states and with DNA damage caused by radiation or chemotherapy.

DNA helicases affect carcinogenesis in a multifaceted way (Figure 5). Impairment in the activity of many DNA helicases correlates with genomic instability and cancer predisposition [3], indicating that helicases prevent chromosomal instability, mutation accumulation, and maintain cellular homeostasis. Numerous genetic diseases that predispose to cancer are linked to mutations in genes encoding helicases. For example, BLM, WRN, and RECQL4 are mutated in Bloom, Werner, and Rothmund-Thomson genomic instability syndromes, respectively, and XPB and XPD are mutated in Xeroderma Pigmentosum, characterized by high risk of skin cancer [36]. Moreover, hereditary mutations and polymorphisms in helicases often predispose, in a heterozygous state, to cancer (e.g. mutations in BLM [37]). On top of that, helicases are frequently mutated in sporadic cancers (Table 1). These suggest that loss of helicase activity promotes genomic instability and triggers tumorigenesis. In parallel, the expression of many DNA helicases is upregulated in cancer cells, enabling the cells to cope with replicative lesions that arise in highly proliferative states and with DNA damage caused by radiation or chemotherapy [3].

### Helicases and estrogen-dependent cancer

Our study reveals that estrogen upregulates the expression of many DNA helicases, which we found to be highly mutated in breast and ovarian cancers (Table 1). Estrogen also drives cells into a proliferating state [10]. Since helicases facilitate the ability of cells to cope with replicative lesions, upregulation of helicases may allow maintenance of genomic stability in estrogen-responding tissues [3]. Therefore, mutations that impair helicase activity may specifically trigger genomic instability in estrogen-responding tissues. Mutations in BLM that result in breast cancer were shown to be in a heterozygous state since the wild-type allele was active in the tumors [38]. Notably, when we examined the state of the helicases found in the screen, in the cosmic database, we found them to be heterozygous in all cases (data not shown). This suggests that for cell survival at least one wild-type allele of the helicase is essential. Based on our results and others we hypothesize that cells, which response to estrogen and harbor a heterozygous mutation in a helicase, may have a better chance for survival due to the estrogen signal because: 1). Estrogen upregulates the expression of the impaired and wild type alleles, which may reduce the mutation effect. 2). Estrogen upregulates the expression of many other helicases, compensating the impairment in the activity of the helicase. 3). Estrogen strongly promotes proliferation. Hence, cells with a mutation in a helicase that respond to estrogen may proliferate. Since helicases have key roles in DNA repair and replication, the mutated helicase may trigger genomic instability in these cells. Our results regarding the interplay between DNA2 and estrogen validate this concept. Mutations in DNA2 found in ovarian cancers impair DNA2 activity (Figure 2) and depletion of DNA2 in estrogen-responding cells (MCF7) ceases proliferation, unless the cells are supplemented with estrogen (Figures 4E and Supplementary Figure S3E). These results demonstrate that cells having a mutation in a helicase can survive if responding to estrogen.

### DSB repair by HR and estrogen-dependent cancer

During HR repair, the 5′ strand resection at DSBs provides 3′ single-stranded DNA that is required for strand invasion and repair. DNA end resection involves the nuclease and helicase activities of the BLM–DNA2–RPA–MRN and EXO1–BLM–RPA–MRN complexes [39]. Notably, apart from RPA, all of these genes, as well as numerous genes involved in HR, were scored in the screen (Table 1 and Supplementary Tables S2 and S3). Genes involved in DSB repair by HR are highly mutated in breast and ovarian cancers [18,19]. Estrogen induces proliferation [10] that may lead to replication stress, which can result in DNA damage [40]. Thus, proper DSB repair mechanisms are essential for maintenance of genomic stability and impairment in HR genes will promote cancer formation. Here we show that many HR genes are upregulated by estrogen (Supplementary Table S1). Moreover, it was suggested that ERα-mediated transcription induces DSBs specifically at estrogen-inducible genes [12]. Thus, accumulation of mutations and small insertions may occur in HR genes, resulting in inaccurate DSB repair.

### Nucleases and estrogen-dependent cancer

Nucleases are essential for genome stability. They act as proof-readers of DNA polymerization during DNA replication. They can remove unusual DNA structures and are involved in DNA repair and in maturation of Okazaki fragments. Incomplete or poorly processed Okazaki fragments can lead to mutations and to the generation of DSBs. The processing of RNA/DNA primers requires both DNA2 and FEN1. DNA2 cleaves long DNA flaps that escape FEN1 activity during Okazaki fragment maturation [41]. Notably, both genes were scored in the screen for DDR genes that are upregulated by estrogen and highly mutated in breast and ovarian cancers (Table 1).

### Future prospectives

Cancer cases consist of a large collection of distinct genetic profiles and the therapeutic strategies designed should consider these differences. Identification of tumor biomarkers has a pivotal role in personalized medicine. These biomarkers may be misregulated expression of genes (overexpressed or repressed) or driver mutations which promote tumorigenesis [42]. We suggest that development of specific inhibitors for nucleases and helicases may facilitate treatment of estrogen-dependent tumors. Inhibition of nucleases and helicases that are overexpressed in a tumor will decrease the growth advantage provided by this overexpression. On the other hand, in cases that the expression or the activity of the nuclease or helicase is reduced, inhibition of other helicases or nucleases may facilitate therapy by simultaneous perturbing the two genes in order to obtain synthetic lethality.

## METHODS

### Microarray data processing and data selection

To identify genes upregulated in the presence of estrogen, expression arrays from the GEO, GDS3217, GDS3283, GDS3285 and GDS3315, (http://www.ncbi.nlm.nih.gov/geo) were analyzed for studies done in MCF7 cell line treated with E2 or mock treated using HG-U133plus2 microarray platforms (Affymetrix). Raw data (CEL files) of all samples were examined with MAS5 probe set condensation algorithm with Expression Console (Affymetrix). Ratio of expression of above 2, between E2-treated and mock treated cells, was considered as an upregulation. A well characterized signature for estrogen response is the upregulation of GREB1, MYB, MYBL1, MYBL2, MYC and PGR [15]. Studies in which at least 5 out of 6 genes of this signature were upregulated in the presence of E2, were considered valid (GDS3285-3h and 6h and GDS3315-24h) [16,17].

In order to filter for genes that have a role in the DDR, the genes upregulated by estrogen were analyzed using the DAVID functional annotation clustering tool (http://david.abcc.ncifcrf.gov; GO_TERM_BP_5;). Genes that clustered as "DNA repair" were selected. David IDs of genes which were not recognized by the GeneCards human gene database (http://www.genecards.org/) were removed.

### Statistical analysis of dbGaP database

In order to test if there is a positive selection for mutations in a gene in cancers, the dbGaP breast and ovarian cancer databases (the database of Genotypes and Phenotypes; http://www.ncbi.nlm.nih.gov/gap) were analyzed for non-silent mutations. The proportion of tumors mutated in a gene in each database was compare to the expected proportion under the assumption for no selection using one-tailed one-proportion z-test. Expected proportion was calculated according to gene size using the evaluation for non silent mutation rate in breast cancer 0.33 per Mb and in ovarian cancer 0.44 per Mb (average of 0.33 and 0.55 per Mb in breast and colorectal cancers, respectively)[20,22]. P-value <0.05 was considered significant.

### shRNA mediated knock-down and real-time PCR

Cells were infected with lentiviruses containing shRNA targeting two sequences of human DNA2 (shDNA2 and shDNA2′) or control (SCR) as described in [31]. 48h flowing infection, cells were selected with puromycin for additional 72h and then used for the experiments. Knockdown efficiencies were tested using real-time PCR with primers for DNA2 (F- 5′ gctgtcctgagtgaaacttttagg 3′ R- 5′ cctcatggagaaccgtacca3′). GAPDH was used as an internal control for housekeeping gene expression (F- 5′ tgagcttgacaaagtggtcg 3′ R- 5′ gctctccagaacatcatcc 3′).

### Cell growth experiments and E2 induction

MCF7 and MDA-MB-435 cells overexpressing GFP (MDA-MB-435/GFP), were maintained at 37°C, 5% CO_2_, with DMEM growth media supplemented with fetal bovine serum (FBS). Prior to testing the response of cells to E2, MCF7 cells were transferred to phenol red free media supplemented with charcoal striped FBS for 72 hours.

To study cellular proliferation, cells were seeded at equal numbers and grown for the indicated time fraims. Cells were fixated with 0.5% glutardialdehyde (Sigma-Aldrich), stained with methylen blue (Sigma-Aldrich), dissolved in 0.1 M boric acid (pH 8.5). Color extraction was performed using 0.1 M hydrochloric acid, and the staining, which is proportional to cell number, was quantified by measuring absorbance at 620 nm.

### Tumor and metastasis assay

Experiments were approved by the Hebrew University Animal Care and Use Committee. For tumor xenografts, 10^6^ MDA-MB-435/GFP cells infected with control or shDNA2′ cells (described above) were mixed with 5 μl matrix gel (25% of final volume, BD Biosciences) and injected into the forth mammary glands bilaterally of 6 weeks old female NOD/SCID mice (6 NOD/SCID female mice when injecting cells infected with shDNA2` and 7 mice when injecting control cells (infected with shSCR). Tumors were measured every 7 days using a caliper and tumor volume was calculated using the modified ellipsoidal formula. 12 weeks from the procedure the mice were sacrificed and tumors and lungs were harvested. Tumors were weighed on an analytic scale. The lungs were analyzed for GFP positive metastasis by florescent microscopy.

### Clinical data of DNA2 in cancers

Kaplan-Meier plots were generated using the online software for genome wide validation of survival associated biomarkers (http://kmplot.com/), 2014 version.

TCGA Provisional databases of Breast Invasive Carcinoma and Uterine Carcinosarcoma were analyzed for gene expression, putative copy-number alterations, and DNA methylation using cBioPortal for Cancer Genomics software (http://cbioportal.org/[35]).

### Yeast assay

Human DNA2 was sub-cloned from pCDNA3-3*Flag-human DNA2 (a kind gift from Jeong-Hoon Kim) to pRS415-GPD using Spe1 and Xho1 restriction enzymes. Then mutagenesis on DNA2 was preformed to generate Q185E, Q203E, E807V and G991S mutants, using site directed mutagenesis. An empty plasmid or plasmids for expressing human DNA2 WT or mutants were introduced into a yeast strain from the Hughes collection [33] were the endogenous promoter of scDNA2 was replaced with a TET-off promoter (*a his3Δ1, leu2Δ0, met15Δ0, ura3Δ0:: URA::CMV-tTA, Kan-tetO7-DNA2*). Yeast cells were grown over-night in a selection liquid media at 30°. Next, the cells were diluted to a concentration equivalent to 1 OD (600 nm), and then diluted serially to 10^−1^, 10^−2^, 10^−3^ and 10^−4^ OD in liquid selection media. 5μl of diluted yeast were seeded on selection plates with or without 20 μg/ml Doxycycline (Dox). Plates were incubated for the indicated hours. UV radiation was of 60 Joule/m^2^ and HU final concentration in media was 100 mM).

For Western blot analysis, yeasts were harvested at logarithmic stage at equal quantities. Proteins were blotted using anti-human DNA2 antibody (Abcam) and anti-yeast G6PD antibody (Sigma-Aldrich) for loading control.

## SUPPLEMENTARY FIGURES AND TABLES




