# Supplementary material for: The DNA2 nuclease/helicase is an estrogen-dependent gene mutated in breast and ovarian cancers
Source: Oncotarget. 2014 Nov 1;5(19):9396–409. doi: 10.18632/oncotarget.2414 (PMC4253442; doi:10.18632/oncotarget.2414)
Supplement: Supplementary file 1 [file oncotarget-05-9396-s001.pdf]

SUPPLEMENTARY FIGURES AND TABLES

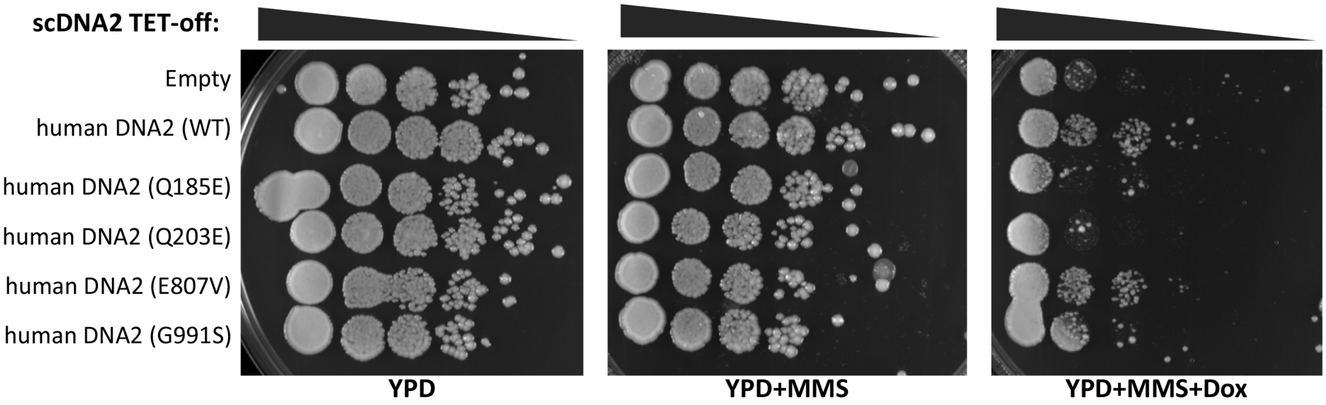

Supplementary Figure S1: Somatic mutations found in cancer cases impair the activity of DNA2. TET-off scDNA2 yeast cells, expressing human DNA2, WT or mutants, or a control vector, were seeded in serial dilutions and grown at 30° for 72 hours. The yeast were seeded on a control plate (YPD), on a YPD plate supplemented with MMS (0.006%; YPD+MMS) or on a YPD plate supplemented with MMS and Dox (YPD+MMS+Dox). Protein expression levels are shown in Figure 2C.

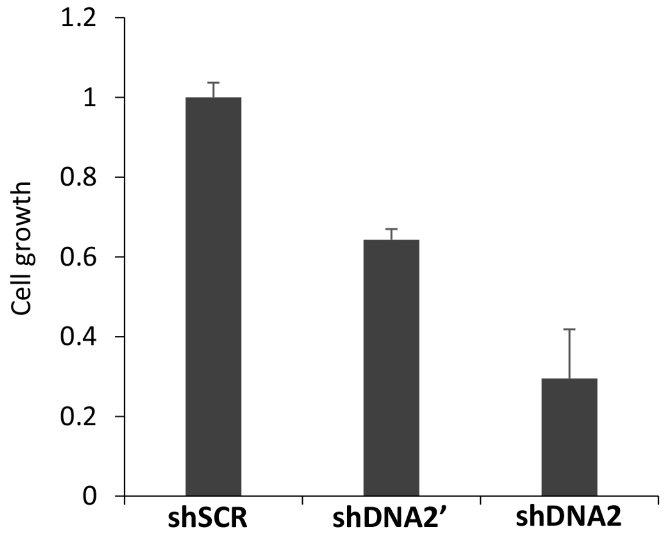

Supplementary Figure S2: Cellular growth of MCF7 cells depleted for DNA2. MCF7 cells infected with shDNA2, shDNA2′ or with a control vector (shSCR), were plated in a 96-well plate at equal numbers. Cellular growth was measured after 72 hours.

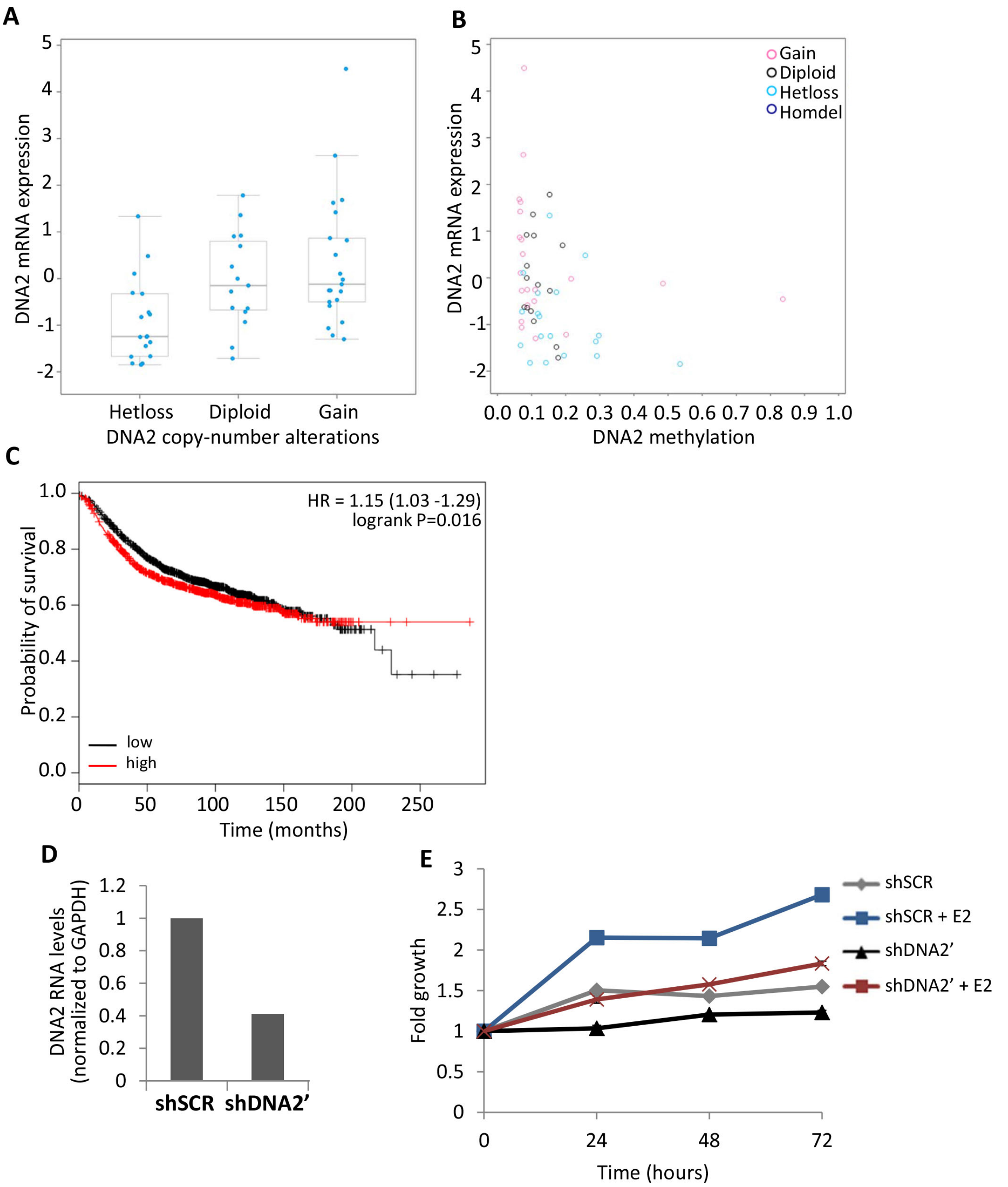

Supplementary Figure S3: Higher DNA2 expression levels correlates with poor prognosis in breast cancer patients. **(A, B)** DNA2 expression levels in 54 uterine carcinosarcomas (TCGA database) were analyzed using cBioPortal, and compared to CNA of DNA2 (A) or to the methylation levels of DNA2 in the same tumors (B). The whiskers in the box plot represent the 10th and the 90th percentile. **(C)** Kaplan-Meier (KM) overall survival curves among 2,878 breast cancer patients according to the levels of DNA2 expression (black curve = cutoff of 50% low expression; red curve = 50% high expression). **(D, E)** MCF7 cells were infected with shDNA2′ or with a control (shSCR), and grown in phenol red-free media with charcoal cleaned serum. Cells were plated in a 96-well plate, 24 h prior to the experiment. E2 (final concentration 10 µM) or ethanol were supplemented at time 0h. Expression levels of DNA2 were tested at time 0 by real-time PCR and normalized by the expression of GAPDH (D). Cells were fixed at indicated time points and cellular growth was measured and compared to day 0 (E).

Supplementary Table S1: List of genes upregulated by estrogen in MCF7 cells. Analysis of expression arrays from the GEO (experiments GDS3285 (3h and 6h time points) and GDS3315 (24h time point)). The ratio in gene expression between cells treated with E2 and mock treated was calculated for each experiment. Indicated in the ratio column is the highest ratio obtained for each gene, and in the study column the experiment that demonstrated this ratio.

Supplementary Table S2: Mutation analysis of DDR genes that are upregulated by estrogen in breast cancer. The genes listed are upregulated by estrogen (Supplementary Table S1) and involved in the DDR according to the DAVID annotation clustering tool. The proportion of tumors harboring a non-silent mutation in these genes was statistically analyzed in the dbGaP breast cancer database. P-values- the probability for receiving the mutation proportion for each gene randomly.

Gene	p-Value
ATR	0.00259
ATRX	1.4E-07
BARD1	0.034299
BLM	<10E-08
BRCA1	<10E-08
BRCA2	<10E-08
BRCC3	0.000177
BRIP1	1.2E-07
CASC5	1E-08
CEP63	0.0232
CHAF1A	0.004019
CHEK1	0.727078
CHEK2	7.01E-05
CLASPIN	0.844589
DNA2	0.09365
DTL	0.27781
ESCO2	0.751368
EXO1	0.04652
FANCA	1E-08
FANCB	0.049008
FANCD2	0.000131
FANCI	0.024622
FEN1	0.094453
GEN1	0.797972
H2AFX	0.003571
JMY	0.389992
MCM7	<10E-08
MCPH1	0.326189
MRE11A	0.000422
MSH2	5.14E-05
MSH6	0.001869
NBN	0.776462

(*Continued*)

Gene	p-Value
NEIL3	0.011997
NUFIP1	0.004242
PCNA	0.672635
PMS1	<10E-08
POLE2	0.174014
POLI	<10E-08
POLK	0.792979
POLQ	9.54E-06
POT1	0.757161
RAD50	0.160438
RAD51	0.000319
RAD51AP1	0.000431
RAD54B	<10E-08
RAD54L	0.029537
RBBP8	3.2E-05
RBL1	0.007913
RECQL	0.016521
RECQL4	0.832126
REV3L	0.000146
RFC2	0.698782
RIF1	0.000102
SETMAR	0.019094
SFPQ	0.023744
SHPRH	0.000538
SLK	0.017327
TIMELESS	0.000693
TIPIN	0.000114
TOP2A	0.22244
TOPBP1	0.000191
TOPORS	0.814687
TP53BP1	0.021345
TYMS	0.06028
UHRF1	0.001069
UNG	0.687862
USP1	0.781066
XRCC5	0.773132
ZMAT3	0.681037

Supplementary Table S3: Mutation analysis of DDR genes that are upregulated by estrogen in ovarian cancer. The genes listed are upregulated by estrogen (Supplementary Table S1) and involved in the DDR according to the DAVID annotation clustering tool. The proportion of tumors harboring a non- silent mutation in these genes was statistically analyzed in the dbGaP ovarian cancer database. P-values- represent the probability for receiving the mutation proportion for each gene randomly.

Gene	p-Value
ATR	0.000131
ATRX	0.315674
BARD1	0.747774
BLM	0.000199
BRCA1	<10E-08
BRCA2	<10E-08
BRCC3	0.0271
BRIP1	0.368221
CASC5	0.847531
CEP63	0.737253
CHAF1A	0.270405
CHEK1	9E-08
CHEK2	<10E-08
CLASPIN	0.809648
DNA2	6.6E-06
DTL	0.741152
ESCO2	0.721398
EXO1	0.014707
FANCA	0.008769
FANCB	0.758598
FANCD2	0.431911
FANCI	0.077515
FEN1	0.04676
GEN1	0.020035
H2AFX	0.612638
JMY	0.774215
MCM7	0.739563
MCPH1	0.755531
MRE11A	0.73797
MSH2	0.262395
MSH6	0.00013
NBN	0.74457

Gene	p-Value
NEIL3	0.133433
NUFIP1	0.702845
PCNA	0.650511
PMS1	0.261659
POLE2	0.101875
POLI	0.007722
POLK	0.238573
POLQ	0.106219
POT1	0.14524
RAD50	0.074764
RAD51	0.033708
RAD51AP1	0.67332
RAD54B	0.764976
RAD54L	0.008102
RBBP8	0.248715
RBL1	0.309739
RECQL	0.151343
RECQL4	0.797437
REV3L	0.723577
RFC2	0.673789
RIF1	0.001279
SETMAR	0.160275
SFPQ	0.174814
SHPRH	0.018964
SLK	0.363913
TIMELESS	0.002747
TIPIN	0.023115
TOP2A	<10E-08
TOPBP1	0.113274
TOPORS	0.000949
TP53BP1	0.003426
TYMS	0.664035
UHRF1	0.74994
UNG	8.52E-06
USP1	0.010362
XRCC5	0.741443
ZMAT3	3.25E-06

Supplementary Table S4: Somatic mutations in DNA2 that occurred in ovarian cancer cases. The dbGAP database was analyzed for mutations in DNA2 gene in ovarian cancers. Position of the mutations in the genome is indicated. WT column- wild type nucleotide. Mutation column- mutation found in ovarian cancer cases. Conserved in column- indicates whether the mutations occurred at a conserved amino-acid residues.

Position in the genome	WT	Mutation	Amino-acid residue substitution	Position in DNA2 protein	Conserved in:		
					Mus musculus	Gallus gallus	S. cerevisiae
chr10;69852350	G	C	Q185E	Nuclease	+	−	−
chr10;69895464	G	C	Q203E	Nuclease	+	+	−
chr10;69888981	T	A	E807V	Helicase	+	+	+
chr10;69846615	C	A	G991C	Helicase	+	+	+
